# Differential regulation of metabolic pathways by androgen receptor (AR) and its constitutively active splice variant, AR-V7, in prostate cancer cells

**DOI:** 10.18632/oncotarget.5585

**Published:** 2015-09-10

**Authors:** Ayesha A. Shafi, Vasanta Putluri, James M. Arnold, Efrosini Tsouko, Suman Maity, Justin M. Roberts, Cristian Coarfa, Daniel E. Frigo, Nagireddy Putluri, Arun Sreekumar, Nancy L. Weigel

**Affiliations:** ^1^ Department of Molecular and Cellular Biology, Baylor College of Medicine, Houston, TX, USA; ^2^ Verna and Marrs McLean Department of Biochemistry, Baylor College of Medicine, Houston, TX, USA; ^3^ Alkek Center for Molecular Discovery, Baylor College of Medicine, Houston, TX, USA; ^4^ Center for Nuclear Receptors and Cell Signaling, Department of Biology and Biochemistry, University of Houston, Houston, TX, USA; ^5^ Genomic Medicine Program, Houston Methodist Research Institute, Houston, TX, USA; ^6^ Scott Department of Urology, Baylor College of Medicine, Houston, TX, USA

**Keywords:** prostate cancer, androgen receptor, splice variant, metabolism, LNCaP

## Abstract

Metastatic prostate cancer (PCa) is primarily an androgen-dependent disease, which is treated with androgen deprivation therapy (ADT). Tumors usually develop resistance (castration-resistant PCa [CRPC]), but remain androgen receptor (AR) dependent. Numerous mechanisms for AR-dependent resistance have been identified including expression of constitutively active AR splice variants lacking the hormone-binding domain. Recent clinical studies show that expression of the best-characterized AR variant, AR-V7, correlates with resistance to ADT and poor outcome. Whether AR-V7 is simply a constitutively active substitute for AR or has novel gene targets that cause unique downstream changes is unresolved. Several studies have shown that AR activation alters cell metabolism. Using LNCaP cells with inducible expression of AR-V7 as a model system, we found that AR-V7 stimulated growth, migration, and glycolysis measured by ECAR (extracellular acidification rate) similar to AR. However, further analyses using metabolomics and metabolic flux assays revealed several differences. Whereas AR increased citrate levels, AR-V7 reduced citrate mirroring metabolic shifts observed in CRPC patients. Flux analyses indicate that the low citrate is a result of enhanced utilization rather than a failure to synthesize citrate. Moreover, flux assays suggested that compared to AR, AR-V7 exhibits increased dependence on glutaminolysis and reductive carboxylation to produce some of the TCA (tricarboxylic acid cycle) metabolites. These findings suggest that these unique actions represent potential therapeutic targets.

## INTRODUCTION

**Figure 1:**
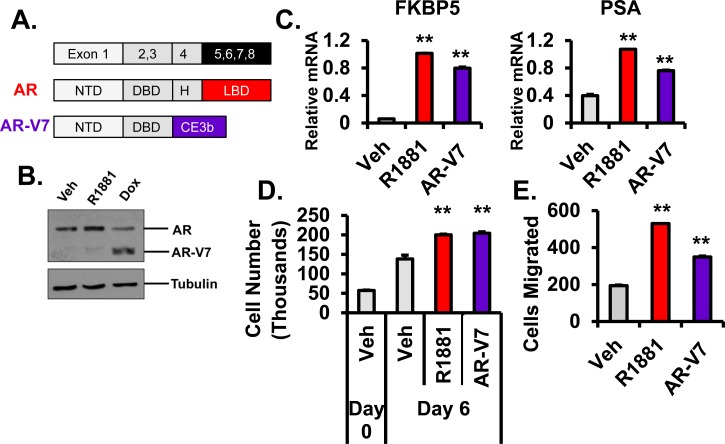
Characterization of AR isoforms **A.** Schematic of full-length androgen receptor (AR) composed of distinct functional domains: amino-terminal transactivation domain (encoded by exon 1), DNA-binding domain (DBD encoded by exon 2 and 3), a hinge region (H encoded by exon 4), and a ligand-binding domain (LBD encoded by exons 4-8). Note that the domains are not proportional to actual size in this diagram. The naturally-occurring V7 splice variant is truncated at exon 3 (amino acids 1-627) followed by 16 unique amino acids. **B.** Inducible LNCaP-AR-V7 cells were changed to stripped serum and treated with vehicle (EtOH), 10 nM R1881, or 20 ng/mL Doxycycline (Dox) for 24 hrs and protein detected by western blot. **C.** LNCaP and LNCaP-AR-V7 cells were changed to stripped serum and treated with vehicle (EtOH), 10 nM R1881, or Doxycycline for 24 hrs and harvested for RNA. AR target gene (*FKBP5* and *PSA*) mRNAs were measured by q-PCR and normalized to *18S* mRNA. **D.** LNCaP-AR-V7 cells were treated with vehicle (EtOH), 1 nM R1881 or 20 ng/ml Dox in stripped serum for the time periods indicated. Cells were counted using a Coulter Counter. **E.** Migration chambers were used to examine migratory ability of the cells. LNCaP-AR-V7 cells were treated with vehicle (EtOH), 1 nM R1881 or 20 ng/mL Dox in serum-free medium (top chamber) and movement into the full-serum medium (bottom chamber) was measured after 48 hours. **p < 0.01 compared to respective vehicle, *n* = 3.

Prostate cancer (PCa) is the most commonly diagnosed non-cutaneous cancer and the second-leading cause of cancer-related death in men in the Unites States [1]. The androgen receptor (AR) plays a critical role both in the development of the normal prostate and in the progression of PCa [2]. AR consists of four functional domains: an amino-terminal transactivation domain (encoded by exon 1), a DNA-binding domain (DBD encoded by exons 2 and 3), a hinge region (H encoded by the 5′ portion of exon 4), and a ligand-binding domain (LBD encoded by the remainder of exon 4 through exon 8) (Figure 1A) [3]. Since AR is the main therapeutic target in PCa, androgen deprivation therapy (ADT) is the first-line of treatment for metastatic disease. Although this therapy is initially successful, tumors recur and are termed castration-resistant prostate cancer (CRPC). CRPC is often characterized by an aberrant reactivation of AR through several mechanisms including expression of constitutively active AR splice variants, which lack hormone binding domains and are thus resistant to ADT [1, 4, 5].

The best-characterized variant is AR-V7 (also termed AR3), which contains exons 1-3 followed by 16 unique amino acids from a cryptic exon 3b [6, 7]. This variant has been detected in CRPC tissue samples and in some cell lines. Although the activities and contributions of variants are still largely unknown, two recent clinical trials show that expression of AR-V7 in tumors correlates with resistance to the anti-androgen, enzalutamide (i.e. MDV3100) and to the CYP17A1 inhibitor, abiraterone, which further reduces levels of androgens [8, 9]. Previous studies have shown that AR-V7 induces PCa cell growth in the absence of androgens, regulates some canonical AR target genes, as well as regulating unique sets of genes [6, 10–12]. However, the biological consequences of unique alterations in gene expression have not been determined and some of these actions may be therapeutic targets.

One known action of AR is to alter metabolism. Several studies have shown that AR signaling stimulates aerobic glycolysis, lipid metabolism, and several anabolic processes in PCa [13–17]. However, there is currently no information on what, if any, role AR-V7 plays in regulating these metabolic pathways. Cancer cells have the ability to alter their cell metabolism to produce compounds to sustain their accelerated growth (Warburg effect) [18]. This phenomenon of metabolic reprogramming has emerged as a hallmark of many cancers [19], and this is a complex, multivariable process.

A majority of metabolic cancer research to date has focused on the role of glycolysis. Increased glycolysis yields more metabolic intermediates to fuel several anabolic processes to produce more building blocks (i.e. amino acids, nucleotides, lipids) for the cells to proliferate rapidly [20]. However, other studies have highlighted the importance of several other key metabolic pathways including the tricarboxylic acid (TCA) cycle and glutamine metabolism (i.e. glutaminolysis) in many cancers [21, 22]. Cancer cells often have increased oxidative phosphorylation (OXPHOS) and elevated uptake and consumption of glutamine [23, 24]. Many cancer cells become addicted to glutamine since it is readily available in high amounts in the circulation and is actively taken up by the cells [25, 26]. Glutamine contributes nitrogen and carbon to many biosynthetic reactions generating lipids and nucleotides. Moreover, glutaminolysis regulates redox homeostasis and modulates the activity of several signal transduction pathways [27, 28].

Previous studies have also integrated metabolic profiling with genomic studies in LNCaP cells to identify transcriptional networks with AR serving as a critical regulator of metabolism [17, 29, 30]. AR regulates key genes involved in cell cycle, glucose metabolism, lipid metabolism, nucleotide metabolism, and amino-acid metabolism [13]. In addition, AR increases glycolysis in PCa cells [13, 16]. To compare the actions of AR and AR-V7, we have employed an inducible AR-V7 model derived from LNCaP cells and have combined steady state metabolomics with metabolic flux studies and gene expression to assess the contributions of AR and AR-V7 to metabolism in PCa cells.

## RESULTS

### AR-V7 induces AR target gene expression, cell growth, and migration

To characterize the functions of AR-V7 in PCa, we generated an LNCaP cell line with doxycycline (Dox) inducible expression of AR-V7 (LNCaP-AR-V7-pHage). AR-V7 lacks the hinge region and LBD found in full-length AR while retaining the NTD and DBD followed by 16 unique amino acids from a cryptic exon 3b (Figure 1A). We induced expression of AR-V7 to similar levels as hormone-stabilized AR (R1881) for our experiments (Figure 1B). Similar to R1881, AR-V7 also induced expression of typical AR target genes, FKBP5 and PSA (Figure 1C). Moreover, R1881 and AR-V7 both increased cell growth and migration (Figure 1D and 1E).

### Both AR isoforms increase glycolysis

**Figure 2:**
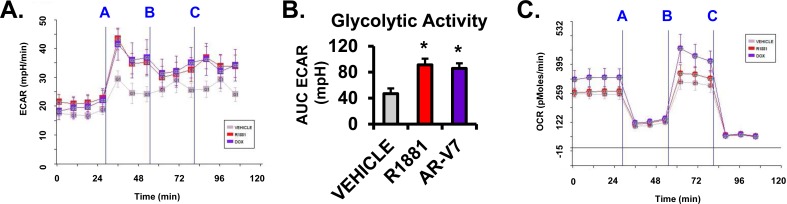
Analysis of AR isoform regulation of ECAR and OCR **A.**-**C.** LNCaP-AR-V7 cells were changed to 10% stripped serum and treated with vehicle (EtOH), 10 nM R1881, or 20 ng/mL Dox for 24 hours prior to Seahorse assay. **A.** Extracellular acidification rate (ECAR), **B.** area under the curve calculations (AUC) for ECAR, and **C.** oxygen consumption rate (OCR) were measured using the Seahorse Bioscience -The XF^e^ Analyzer. Values were normalized to cell number. Cells were treated with different mitochondrial inhibitors (i.e. **A.** Oligomycin, **B.** FCCP, and **C.** Rotenone) over the course of the flux experiment. Oligomycin is a Complex V inhibitor that blocks ATP synthase. FCCP is a mitochondrial oxidative phosphorylation inhibitor. Rotenone is a Complex I inhibitor that blocks electron flow. Values were normalized to cell number. *n* = 3.*p < 0.05 compared to respective vehicle, *n* = 3.

Previous studies have shown that activation of AR with R1881 increased glycolysis [13, 16]. To determine whether AR-V7 also increases glycolysis, a Seahorse assay was done to measure ECAR (extracellular acidification rate), a measure of glycolysis. Similar to R1881 treatment, induction of AR-V7 increased ECAR (Figure 2A and 2B). The isoforms respond similarly with respect to ECAR when treated with the mitochondrial inhibitors, FCCP and rotenone. In parallel, although there was a trend for increased OCR (oxygen consumption rate) in AR-V7 expressing cells, there was no significant difference for either isoform relative to vehicle (Figure 2C) consistent with results of an earlier AR study by Massie et al., 2011 [13].

### AR and AR-V7 differentially regulate steady state levels of metabolites

**Figure 3:**
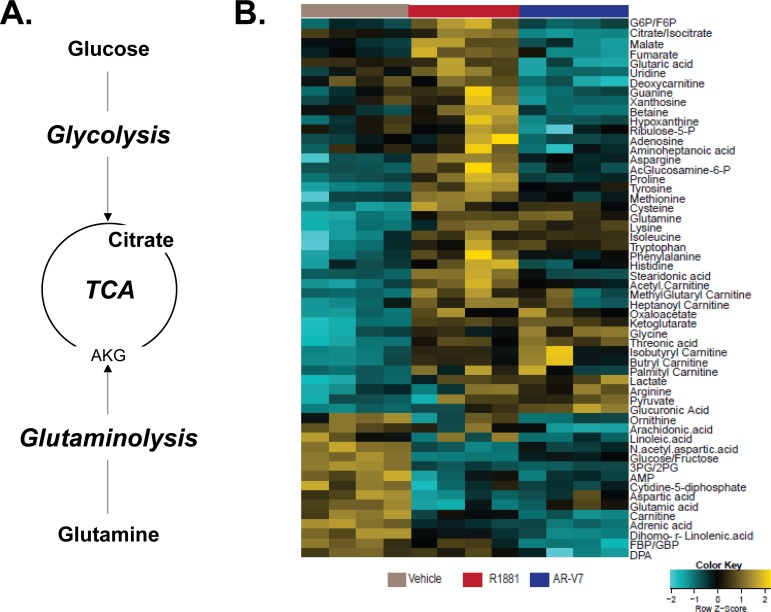
Full-length AR and AR-V7 have unique metabolic profiles in LNCaP cells **A.** A simplified diagram of metabolism highlighting the basic pathways of glycolysis, the TCA cycle, and glutaminolysis. **B.** Using liquid chromatography-mass spectrometry (LC-MS), we examined the effect of AR or AR-V7 activation on the levels of a series of metabolites. In this study, the vehicle group is parental LNCaP cells treated with EtOH and 20 ng/mL Dox. The R1881 group is parental LNCaP cells treated with 10 nM R1881 and 20 ng/mL Dox. The AR-V7 group is inducible LNCaP-AR-V7 cells treated with 20 ng/mL Dox. All treatments were for 48 hours. The metabolic profile of Vehicle versus R1881 (i.e. activated AR) versus AR-V7 (20 ng/ml Dox treated cells) is depicted. G6P/F6P = glucose-6-phosphate / fructose-6-phosphate, 3PG/2PG = 3-phosphoglycerate / 2-phosphoglcerate, AMP = adenosine monophosphate, FBP/GBP = fructose-bisphosphate / glucose-bisphosphate, and DPA = docosapentaenoic acid.

**Figure 4:**
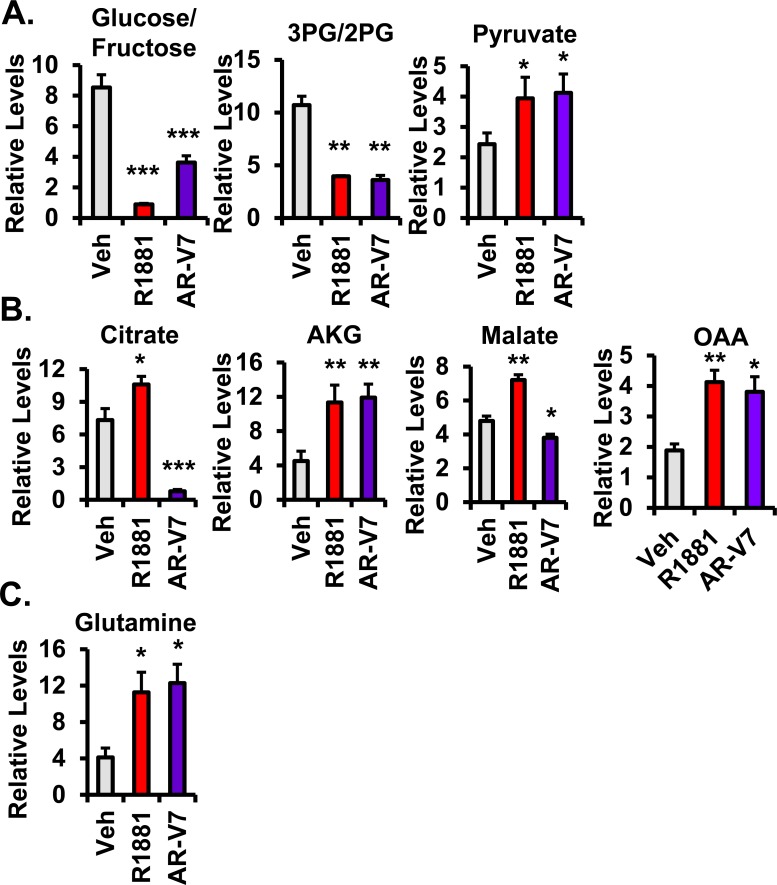
AR and AR-V7 differentially regulate metabolites in LNCaP cells AR isoform-specific regulation of specific metabolites involved in **A.** glycolysis, **B.** the tricarboxylic acid (i.e. citric acid) cycle (TCA), and **C.** glutaminolysis are depicted from the following treatments: vehicle (EtOH), 10 nM R1881, and 20 ng/mL Dox (i.e. AR-V7 expressing cells). *p < 0.05, **p < 0.01, and ***p < 0.001 compared to respective vehicle, *n* = 4. 3PG/2PG = 3-phosphoglycerate / 2-phosphoglcerate, AKG = α-ketoglutarate, and OAA = oxaloacetate.

**Figure 5:**
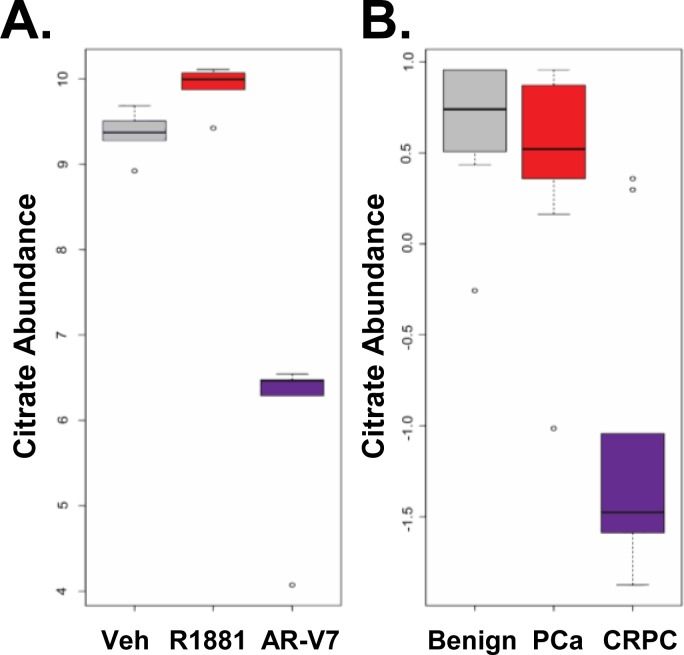
Castration resistant tumors have reduced levels of citrate The relative citrate levels in the samples from Figure 4B were compared with a data set [17] measuring citrate in 16 benign prostate tissue samples, 12 androgen-dependent prostate cancer tumor samples, and 14 metastatic, castration-resistant prostate cancer tumor samples. **A.** The steady-state levels of citrate from the LNCaP and LNCaP-AR-V7 cells treated with vehicle, 10 nM R1881, and 20 ng/mL Dox (i.e. AR-V7 expressing cells) are graphed as boxplots from our metabolomics data set (Figure 4B). **B.** Citrate levels from the tumor samples are depicted from benign, PCa, and CRPC tissues samples.

A simplified diagram of metabolism in Figure 3A, shows that cells utilize both glucose through glycolysis to produce pyruvate and glutamine through glutaminolysis to produce α-ketoglutarate, both of which can feed the TCA cycle to generate precursors essential for other key biochemical pathways. To more broadly assess the effects of AR and AR-V7 on metabolism, steady state levels of approximately 100 metabolites including glycolytic intermediates, TCA cycle components, amino acids, and some nucleotides were measured using liquid chromatography-mass spectrometry (LC-MS). A heat map of the metabolites regulated by one or both of the AR isoforms revealed that AR and AR-V7 regulate a majority of the metabolites in a similar manner although not always to the same extent (Figure 3B). However, there were some key differences particularly in the TCA cycle. Consistent with both isoforms stimulating glycolysis, both AR and AR-V7 decreased glucose/fructose and 3PG/2PG (3-phosphoglycerate, 2-phosphoglycerate) levels, but increased pyruvate levels (Figure 4A). In contrast, AR and AR-V7 differentially regulated levels of the TCA cycle metabolites. AR increased the steady state levels of all of the TCA cycle intermediates while AR-V7 decreased levels of some metabolites but increased the levels of others (Figure 4B). The alterations in the steady state levels of citrate were particularly striking with hormone treatment increasing the levels of this metabolite while AR-V7 dramatically reduced citrate levels. In contrast, α-ketoglutarate (AKG) and oxaloacetate (OAA) were increased by both AR-V7 and AR. Furthermore, both isoforms increased the steady state levels of glutamine (Figure 4C) and AKG (Figure 4B) suggesting potential regulation of glutaminolysis. The striking difference observed in the *in vitro* citrate levels is consistent with patient tumor derived metabolomics data. Citrate levels are high in the normal prostate and in androgen-dependent PCa but are low in CRPC tumors, which frequently express AR-V7 (Figure 5A and 5B).

### Regulation of glucose flux by AR and AR-V7

**Figure 6:**
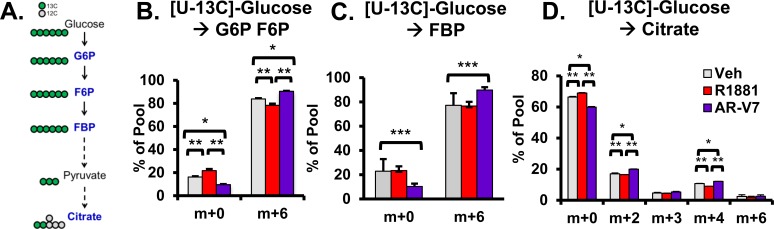
Analysis of AR isoform-specific progression through glycolysis with [U-13C]-glucose metabolic flux analysis **A.** Schematic of universally-labeled glucose with 13C (green circles) progressing through glycolysis and entering the TCA cycle with citrate having 2 carbons labeled and 4 unlabeled carbons (grey circles) in the first round. The mass isotopomers of the metabolites labeled in blue were analyzed. **B.** Mass isotopomers of glucose-6-phosphate and fructose-6-phosphate (G6P F6P) **C.** fructose-1,6-bisphosphate (FBP), and **D.** citrate were measured. First, LNCaP and LNCaP-AR-V7 cells were changed to 10% stripped serum and treated with vehicle (EtOH), 10 nM R1881, and/or 20 ng/mL Dox for 24 hours. The next day the medium was changed to starvation medium (i.e. without glucose for 6 hours). Then [U-13C]-glucose was added to each group for 3 hours. Cells were harvested and processed as described in the methods. *p < 0.05, **p < 0.01, *n* = 4.

Changes in steady state levels of metabolites may be caused by reduced synthesis and/or increased utilization. To distinguish these possibilities, flux analyses were performed. [U-13C]-glucose labeled metabolic flux analysis was employed to analyze AR isoform-specific progression through glycolysis. In this labeling scheme depicted in Figure 6A, glucose-derived glucose-6-phosphate (G6P), fructose-6-phosphate (F6P), and fructose-1,6-bisphosphate (FBP) were detected as an m+6 isotopomer retaining all six labeled carbons. Citrate is detected as multiple isotopomers (m+2, m+3, m+4, and m+6) indicating flow of glucose-derived carbon through multiple rounds of glycolysis. After 3 hours of [U-13C]-glucose labeling, isotopomer analysis revealed that glucose consumption via glycolysis was increased in AR-V7 expressing cells. In particular, expression of AR-V7 corresponded to a statistically significant increase in glycolytic intermediates: G6P F6P (m+6) and FBP (m+6) as well as elevated incorporation of glucose-derived carbon pool into citrate (m+2, m+4) (Figure 6B, 6C, and 6D). The AR-V7 mediated increase in 13C-labeled citrate metabolites suggests that reduced levels of this metabolite seen using steady state metabolomics is likely a result of increased utilization of citrate rather than a perturbation in its synthesis.

**Figure 7:**
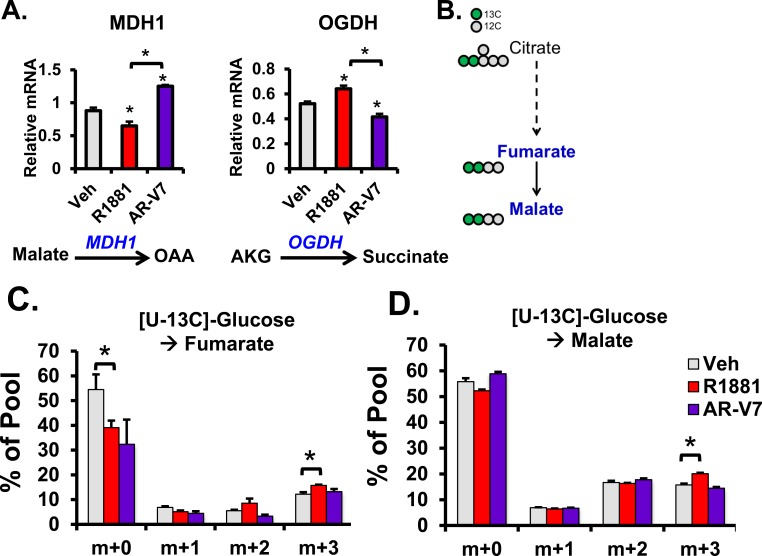
Comparison of AR and AR-V7 Induced flux through the TCA cycle in LNCaP cells **A.** LNCaP and LNCaP-AR-V7 cells were changed to 10% stripped serum and treated with vehicle (EtOH), 10 nM R1881, or 20 ng/mL Dox for 24 hrs and harvested for RNA. Metabolic genes, MDH1 and OGDH, were measured by q-PCR and normalized to 18S mRNA. **B.** Schematic of universally-labeled glucose with 13C (green circles) entering and progressing through the TCA cycle with citrate having 2 labeled carbons and 4 unlabeled carbons (grey circles) at the start of the first cycle. The mass isotopomers of the metabolites labeled in blue were analyzed. Mass isotopomers of **C.** fumarate and **D.** malate were measured after culture of LNCaP and LNCaP-AR-V7 as described in Figure 6. *p < 0.05, **p < 0.01, *n* = 4.

All of the measured TCA cycle intermediates were elevated by AR in our steady state metabolomics analysis. Whereas citrate, α-ketoglutarate (AKG), fumarate, malate, and oxaloacetate (OAA) were increased by AR, only AKG and OAA were increased by AR-V7 (Figure 3B and 4B). Analysis of the mRNAs for the enzymes responsible for the conversion of AKG to succinyl-CoA and CO_2_ [31] and the conversion of malate to OAA [32] revealed that they are differentially regulated by the AR isoforms. Hormone treatment to activate AR decreased levels of malate dehydrogenase 1 (MDH1) and increased levels of α-ketoglutarate dehydrogenase (OGDH) (Figure 7A). This favors the accumulation of malate and fumarate, respectively. Conversely, AR-V7 increased levels of MDH1 and decreased OGDH. This gene regulation is consistent with the steady-state metabolomics data. Increased MDH1 supports increased conversion of malate to OAA, which was high in AR-V7 expressing cells. Moreover, decreased OGDH supports decreased conversion of AKG whose levels are also high in AR-V7 cells.

We next used [U-13C]-glucose metabolic flux analysis to examine AR isoform-specific progression through the canonical TCA cycle. In this labeling scheme depicted in Figure 7B, TCA cycle intermediates could be detected through several cycle progressions. In the first round, the TCA cycle would begin with two carbons of citrate labeled and progress through the cycle ending with late TCA cycle intermediates (i.e. malate and fumarate) also having two carbons labeled. These are detected by the mass spectrometer as m+2 isotopomers. The subsequent cycles would produce m+3 and then m+4 isotopomers. After 3 hours of [U-13C]-glucose labeling, isotopomer analysis revealed that glucose utilization via the TCA cycle was increased in hormone treated but not in AR-V7 expressing cells. In particular, R1881 increased levels of late TCA cycle intermediates: fumarate (m+3) and malate (m+3) (Figure 7C and 7D). Although Tennakoon et al. [16] have previously reported increased steady state levels of TCA metabolites in response to R1881, to our knowledge this is the first analysis showing R1881 accelerated flux through the canonical TCA cycle.

### AR-V7 enhances glutamine metabolism via reductive carboxylation

**Figure 8:**
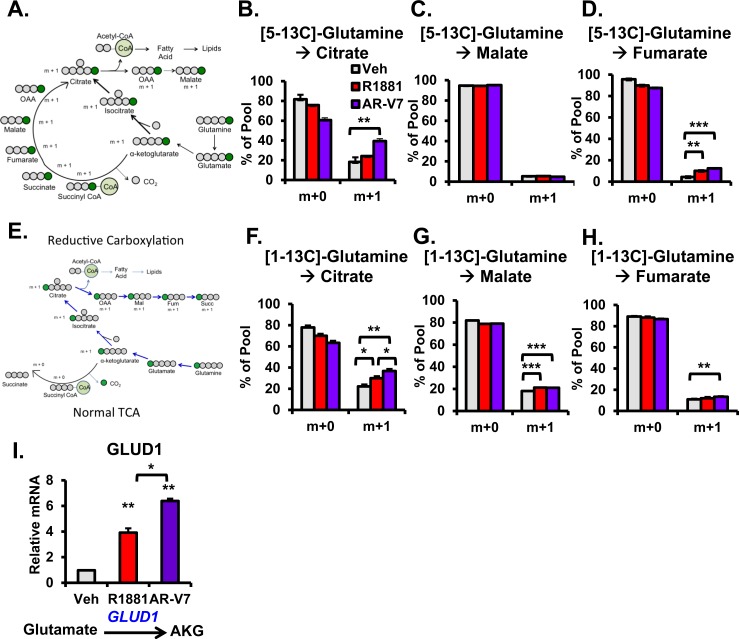
Analysis of AR isoform-specific utilization of glutaminolysis with [5-13C]-glutamine and [1-13C]-glutamine metabolic flux analysis **A.** Schematic of [5-13C]-glutamine (i.e. only the fifth carbon of glutamine is labeled; 13C (green circles)) progressing through either the TCA cycle or reductive carboxylation leading to a production of citrate having 1 carbon labeled and 4 unlabeled carbons (gray circles) at the end of the first cycle. Mass isotopomers of **B.** citrate **C.** malate, and **D.** fumarate were measured. LNCaP and LNCaP-AR-V7 were changed to 10% stripped serum and then treated with vehicle (EtOH), 10 nM R1881, and/or 20 ng/mL Dox for 24 hours. The next day the medium was changed to starvation medium (i.e. without glutamine for 6 hours). Then [5-13C]-glutamine was added to each group for 3 hours. Cells were harvested and processed as discussed in the methods. **E.** Schematic of [1-13C]-glutamine (i.e. only the first carbon of glutamine is labeled; 13C (green circles)) progressing through reductive carboxylation with citrate having 1 carbon labeled and 4 unlabeled carbons (gray circles) at the end of the first cycle. Mass isotopomers of **F.** citrate **G.** malate, and **H.** fumarate were measured after culture of LNCaP and LNCaP-AR-V7 as described for [5-13C]-glutamine above. **I.** LNCaP-AR-V7 cells were changed to 10% stripped serum and then treated with vehicle (EtOH), 10 nM R1881, or 20 ng/mL Dox for 24 hours. Metabolic gene involved in glutaminolysis, GLUD1, was measured by q-PCR and normalized to 18S mRNA. *p < 0.05, **p < 0.01, and ***p < 0.001 compared to respective vehicle, *n* = 4.

The TCA cycle generates several metabolic intermediates that feed multiple biosynthetic pathways to produce lipids, nucleic acids, and proteins essential for cell growth [33]. However, in many cancer cells, glutamine metabolism is utilized to generate energy and metabolic intermediates for the rapidly growing cells [34, 35]. Therefore, we examined AR isoform-specific metabolic flux through canonical and reductive carboxylation of glutamine. [5-13C]-glutamine and [1-13C]-glutamine metabolic flux analyses were employed to analyze AR isoform-specific utilization of reductive carboxylation (Figure 8A). When using [5-13C]-glutamine, the fifth carbon of glutamine is labeled. Thus, the m+1 isotopomers of the TCA cycle intermediates can be derived through either the canonical TCA cycle or reductive carboxylation. Consistent with this, after 3 hours of [5-13C]-glutamine labeling, isotopomer analysis revealed that both AR and AR-V7 significantly increased the m+1 isotopomer of fumarate (Figure 8D). Additionally, AR-V7 significantly increased the levels of citrate (m+1) (Figure 8B). This suggests that both receptors can generate these metabolites either through the canonical TCA cycle or from utilization of reductive carboxylation. In order to further understand the extent of utilization of glutamine-derived carbon via canonical versus reductive carboxylation routes, we performed [1-13C]-glutamine metabolic flux analyses. Here, only the first carbon of glutamine is labeled (Figure 8E) and thus, the intermediates generated by canonical functioning of the TCA cycle will remain unlabeled (since the first carbon of AKG is lost as CO_2_ during its conversion to succinyl CoA). In contrast, if the cells utilized reductive carboxylation, then the mass spectrometer would detect m+1 isotopomers for the TCA intermediates. Interestingly, after 3 hours of [1-13C]-glutamine labeling, AR and AR-V7 significantly increased m+1 isotopomers of citrate and malate compared to vehicle control (Figure 8F and 8G). Additionally, compared to AR, AR-V7 increased levels of citrate and fumarate (m+1) (Figure 8F and 8H) suggesting increased utilization of reductive carboxylation of glutamine by this AR isoform. Both AR and AR-V7 significantly increased glutamate dehydrogenase (GLUD1), a mitochondrial matrix enzyme that catalyzes the oxidative deamination of glutamate to AKG and ammonia (Figure 8I) [36]. AR-V7 more robustly increased GLUD1 than hormone treatment consistent with increased reductive carboxylation.

### AR preferentially induces lipid accumulation

**Figure 9:**
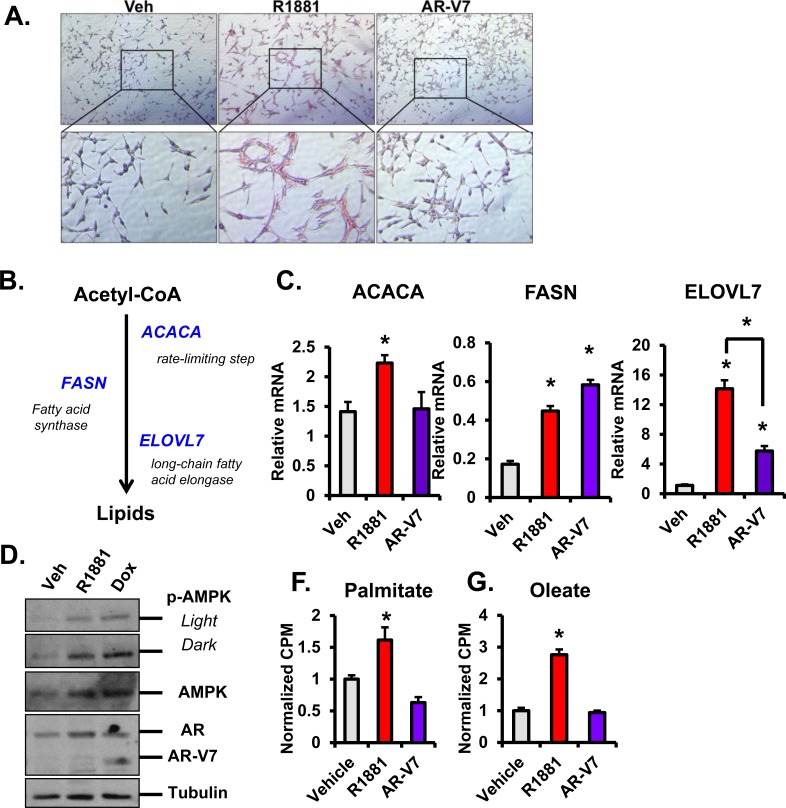
AR induces lipid accumulation and beta-oxidation and regulates the fatty acid synthesis pathway **A.** LNCaP-AR-V7 cells were changed to 10% stripped serum and then treated with vehicle (EtOH), 10 nM R1881, or 20 ng/mL Dox for 6 days. Media and treatments were changed out and replenished after 3 days. Oil Red O staining was used to stain for lipids *in vitro*. Red staining indicates the presence of lipid droplets. Images were taken at 20X magnification. **B.** Schematic of fatty acid synthesis pathway depicting the generation of lipids through conversion of Acetyl CoA through the rate-limiting step of ACACA, FASN, and the downstream long-chain fatty acid elongase, ELOVL7. **C.** LNCaP and LNCaP-AR-V7 cells were treated with vehicle (EtOH), 10 nM R1881, or 20 ng/mL Dox for 24 hours and harvested for RNA. ACACA, FASN, and ELOVL7 mRNAs were measured by q-PCR and normalized to 18S mRNA. **D.** LNCaP-AR-V7 cells were changed to 10% stripped serum and treated with vehicle (EtOH), 10 nM R1881, or 20 ng/mL Dox for 72 hours. Cells were harvested and AR, AR-V7, p-AMPK, AMPK, and Tubulin protein expression was detected with Western blot. (E-F) LNCaP and LNCaP-AR-V7 cells were changed to 10% stripped serum and then treated with vehicle (EtOH), 10 nM R1881, or 20 ng/mL Dox for 24 hours. Radiolabeled palmitate or oleate was added to the media. Oxidation of radiolabeled **E.** palmitate and **F.** oleate were measured using CO_2_ trap assays and values normalized to DNA content. *p < 0.05 compared to respective vehicle, *n* = 3.

Although the role of glucose is the primary focus of many metabolic studies, several groups have shown the importance of other metabolites in PCa including fatty acids [37, 38]. AR has been reported to induce fatty acid synthase (FASN) [13] and to cause lipid droplet accumulation [39, 40]. To determine whether AR-V7 also causes a similar lipid droplet accumulation, cells were treated with vehicle, 10 nM R1881, or 20 ng/mL Dox (AR-V7) for six days and stained with Oil Red O. While androgen-mediated AR activity increased lipid accumulation in LNCaP cells as expected, AR-V7 did not induce a similar accumulation (Figure 9A). Consistent with this, a comparison of the expression of genes in the fatty acid synthesis pathway (Figure 9B) showed that AR significantly increased mRNA expression of the rate-limiting enzyme, ACACA while AR-V7 did not (Figure 9C). In contrast, both isoforms induced FASN (fatty acid synthase) and ELOV7 (i.e. a long chain fatty acid elongase). The difference in lipid droplet accumulation suggests either AR-V7 does not effectively induce synthesis of one or more of the components of lipid droplets, or that the synthesized lipids are used for other purposes such as phospholipid synthesis. Although this pathway was not the major focus of our study, ACACA activity is inhibited by phosphorylation by AMPK [41] and AR activates AMPK [16]. To test whether AR-V7 also induces this pathway, AMPK and p-AMPK were measured after activation of AR or AR-V7. Both AR isoforms increased expression of p-AMPK and AMPK similar to what has been previously reported [16] (Figure 9D). These findings suggest that AR and AR-V7 should reduce ACACA activity. However, citrate allosterically activates ACACA overcoming the inhibitory effects of phosphorylation [42]. Therefore, the elevated levels of citrate in hormone treated cells likely counteract the inhibitory phosphorylation of AMPK on ACACA.

Lipids also can be metabolized through β-oxidation to provide energy and previous studies have shown that AR increases β-oxidation [16]. To determine whether AR-V7 also stimulates β-oxidation of exogenously added radiolabeled palmitate or oleate, assays were performed as previously described [16] and radiolabeled CO_2_ measured. In LNCaP cells, hormone treatment induced oxidation of radiolabeled palmitate and oleate (Figure 9E and 9F) consistent with previous reports [16]. However, AR-V7 did not induce oxidation of either exogenously added fatty acid. These findings are consistent with the data presented in Figure 9A indicating that AR-V7 does not alter fatty acid metabolism in the same manner as full-length AR.

**Figure 10:**
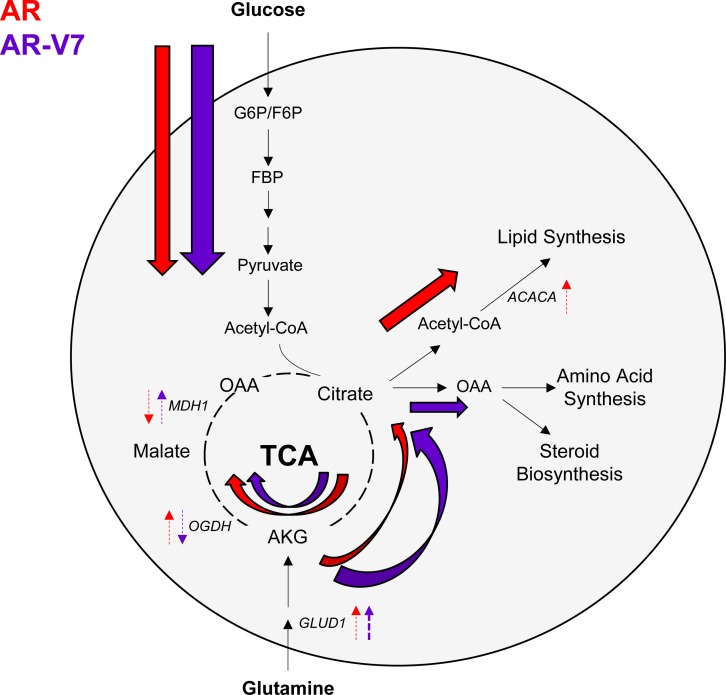
AR utilizes the TCA cycle, while AR-V7 preferentially enhances glutaminolysis This model summarizes our findings of the metabolic profiles and functions of AR and AR-V7. Beginning with glycolysis, both AR and AR-V7 increased extracellular acidification rate measuring glycolytic activity. Glycolytic genes and intermediate metabolites were also increased by both AR isoforms. Metabolic flux analysis revealed that AR-V7 accelerated glycolysis more effectively than AR. Steady state metabolites, gene expression, and metabolic flux studies showed that AR progresses through the TCA cycle. Dashed arrows next to the metabolic genes indicate AR isoform specific action. AR can utilize both glucose and glutamine as starting material to progress through the TCA cycle to generate energy and fuel for cell growth and survival. Furthermore AR can utilize both the canonical the TCA cycle and reductive carboxylation for glutamine metabolism. Interestingly, AR-V7 has an increased dependence on reductive carboxylation to utilize glutamine as an energy source as seen with increased gene expression and metabolic flux analysis. Thus, AR-V7 can mimic AR's metabolic functions regulating glycolysis and also has unique metabolic roles preferentially enhancing glutaminolysis often seen in many cancer cells (i.e. a component of the Warburg effect).

Overall, this study is the first to show the unique metabolic profiles and functions of AR-V7 summarized in the model (Figure 10). Both AR and AR-V7 increase glycolysis through induction of several intermediate metabolites. Intriguingly, AR-V7 further enhanced glycolytic flux more effectively than AR at early time points and showed enhanced conversion of glutamine to citrate via reductive carboxylation. Furthermore, decreased steady state levels of citrate despite an enhanced rate of its synthesis from glucose and glutamine, implies an increase in the utilization of this key TCA intermediate to generate biochemical components required for CRPC progression.

## DISCUSSION

CRPC is characterized by an aberrant reactivation of AR through several mechanisms including AR mutations, AR amplifications, altered signaling pathways, aberrant local synthesis of androgens, and expression of constitutively active AR splice variants [1]. Although AR-V7 is the most thoroughly studied variant, many aspects of its activities and function are still unknown. A major debate in the field is whether AR-V7 is simply a constitutively active partial substitute for full-length AR or whether it has its own unique gain-of-function activities. In order to investigate these questions, we developed an inducible LNCaP PCa cell line to regulate expression of AR-V7. We induced AR-V7 to similar levels as hormone-treated (i.e. activated) AR (Figure 1B) to directly compare the actions of AR and AR-V7. Although AR-V7 is expressed at lower levels in many tumors there is also evidence that it can be expressed at levels similar to AR levels found in hormone dependent tumors [43, 44]. In this model, AR-V7 induced classical target genes (PSA and FKBP5) and increased cell growth and migration similar to AR (Figure 1C, 1D, and 1E). Thus, with this cell model we confirmed that AR-V7 mimics some aspects of AR function as expected based on previous studies.

A number of studies have shown that activated AR alters cell metabolism by stimulating aerobic glycolysis, lipid metabolism, and several anabolic processes in PCa [13–15]. Thus, we sought to compare the actions of the AR isoforms in altering metabolism and, in particular, focused on their capacity to utilize glucose and glutamine, two essential energy sources for cells. The isoforms stimulated glycolysis to a similar extent when measured by ECAR, which is a reflection of lactate production (Figure 2). However, an analysis of the steady state levels of a broad range of metabolites clearly showed that there were major isoform specific differences. If AR-V7 were simply a partial substitute of AR, we would expect that the changes in metabolites would be in the same direction for AR-V7 and AR although the magnitude could differ. While this is true for many metabolites (Figure 3B), there are a number of metabolites that are regulated in the opposite direction by the two isoforms or exclusively by one isoform.

One of the most striking differences is the change in citrate levels (Figure 4B). Whereas AR increased citrate, a metabolite typically found in high concentrations in androgen-dependent prostate tissue (both normal and PCa), AR-V7 strongly reduced the level of citrate. Interestingly, reduced citrate levels are a characteristic of CRPC, which frequently express AR-V7 (Figure 5B). Reduced citrate levels could be a reflection of a failure to synthesize citrate and/or enhanced metabolism of citrate. To distinguish between the two possibilities, a 3-hour flux analysis was first performed using [U-13C]-glucose to measure glycolytic and TCA metabolites. AR-V7 expression enhanced the levels of citrate derived from the labeled glucose (Figure 6D), indicating that there is no reduction in the capacity of this AR isoform to produce citrate derived from glucose. The second major potential source to generate citrate is through metabolism of glutamine. Flux analyses performed using [5-13C]-glutamine showed that AR-V7 enhanced the overall production of labeled citrate derived from glutamine either through the canonical TCA cycle or through reductive carboxylation (Figure 8B) or both. The increased use of reductive carboxylation by AR-V7 to generate the citrate pool was verified using flux studies employing [1-13C]-glutamine (Figure 8F). Thus, AR-V7 accelerates synthesis of citrate, but the low steady state levels suggest that it also enhances subsequent metabolism of citrate.

The alteration in steady state levels of citrate is not the only AR-V7 specific change. Whereas AR increased the levels of all TCA metabolites, there was a marked decrease in malate as well as citrate in AR-V7 expressing cells despite elevated levels of AKG and OAA. We identified two factors that are likely to contribute to the reduced levels of malate despite enhanced levels of AKG and OAA. First is the differential regulation in the canonical TCA cycle examined through regulation of key metabolic genes and with the use of glucose flux analysis. For example, AR-V7 increased levels of MDH1, which would be predicted to result in lower levels of malate and higher levels of OAA. Moreover, AR-V7 decreased levels of OGDH suggesting higher levels of AKG in AR-V7 expressing cells relative to vehicle cells (Figure 7A). Consistent with this is the observation that the glucose flux studies showed enhanced production of labeled fumarate and malate in response to R1881, but not to expression of AR-V7 (Figure 7C and 7D) further indicating full-length AR's role in progressing through the canonical TCA cycle. The second factor contributing to differential levels of malate by the two receptors is their role in the reductive carboxylation pathway. The glutamine flux studies showed that AR-V7 expression increased usage of glutamine to produce TCA cycle metabolites. Interestingly, the [1-13C]-glutamine flux indicated that this enhancement was partially mediated through an increase in reductive carboxylation of glutamine (Figure 8).

The reduced steady state levels of citrate in AR-V7 cells despite synthesis of citrate suggest that it is rapidly metabolized to other compounds needed for cell growth. Among the possibilities are lipid and phospholipid synthesis, amino acid synthesis, and synthesis of steroids. Since AR is known to induce lipid accumulation [39, 40], we asked whether AR-V7 similarly induced lipid droplet accumulation. We found that AR-V7 does not induce lipid accumulation despite inducing FASN mRNA to similar levels as R1881 treated cells (Figure 9). Neutral lipids can be used for many purposes including synthesis of phospholipids, post-translational modification of proteins, and β-oxidation. Our studies showed, that in contrast to AR [16], AR-V7 does not accelerate β oxidation measured using exogenous radiolabeled lipids (Figure 9F and 9G). Whether AR-V7 stimulates another pathway to utilize lipids remains to be determined.

ACACA is typically the rate-limiting step in fatty acid synthesis. There was a small induction of ACACA mRNA by R1881, but not by AR-V7. ACACA is inhibited by AMPK mediated phosphorylation [41]. Frigo's group has shown that full-length AR increases phosphorylation (activation) of AMPK [16]. The level of AMPK phosphorylation induced by AR-V7 was comparable to that induced by AR (Figure 9D) indicating that preferentially enhanced phosphorylation of AMPK is not responsible for the lack of lipid accumulation. Since citrate is an allosteric regulator of ACACA [42], it is possible that the higher levels of citrate in AR-dependent cells combined with the increase in ACACA mRNA contribute to higher levels of ACACA activity in cells with activated AR relative to AR-V7 expressing cells.

Both AR and AR-V7 increase levels of OAA, which can be used to make other TCA metabolites or as a substrate to make amino acids. OAA can be derived from malate in the canonical TCA cycle or from citrate through reductive carboxylation. The AR-V7 flux analyses suggest that AR-V7 is making citrate both from glucose and through reductive carboxylation of glutamine. Synthesis of OAA from citrate also produces acetyl-CoA, which can be used for lipid synthesis or for cholesterol synthesis, which ultimately leads to the production of steroids. A reduced level of ACACA may favor cholesterol/steroid synthesis in cells expressing AR-V7, but this remains to be tested experimentally.

In summary, we conclude AR-V7 mimics AR in regulating some of the canonical AR target genes, cell growth and migration, and in inducing glycolysis. Our study is the first to identify the novel metabolic functions of AR-V7. Similar to AR, AR-V7 accelerated glycolysis, but differences were detected in the subsequent synthesis/usage of these metabolites. Furthermore, steady state and metabolic flux analyses demonstrated that AR induced flux through both the canonical TCA cycle and reductive carboxylation of glutamine. In contrast, AR-V7 enhanced reductive carboxylation to generate citrate from glutamine. Furthermore, the changes in levels of metabolic genes (OGDH, MDH1, and GLUD1) were consistent with these findings, but regulation may also occur at additional levels. This study suggests that AR-V7 does not simply substitute for AR, but exhibits gains of function that may include the ability to grow more efficiently in an oxygen poor environment. Lastly, this study yields potential pathways to target in combination with current CRPC therapies to better treat patients whose tumors express AR-V7. For example, AR-V7's reliance on glutamine suggests the use of a glutaminase inhibitor could be beneficial although additional pre-clinical studies are needed to determine whether AR-V7 tumors are strongly dependent on glutamine. Calithera is currently testing a glutaminase inhibitor, CB-839, in a phase I clinical trial in solid tumors that does not include PCa. If AR-V7 tumors are dependent on glutamine, then CRPC should be considered in subsequent trials [45, 46].

## MATERIALS AND METHODS

### Cell culture and supplies

LNCaP cells were purchased from the American Type Culture Collection (Manassas, VA). The LNCaP-AR-V7-pHage cell line is an LNCaP cell line with TET (doxycycline) regulated AR-V7 expression generated in our lab [11]. Parental LNCaP and LNCaP-AR-V7-pHage cells were maintained RPMI and 10% serum as previously described [11, 47]. Methyltrienolone (R1881) was purchased from Perkin-Elmer (Boston, MA). [U-13C]-glucose, [1-13C]-glutamine, and [5-13C]-glutamine were purchased from Cambridge Isotope Laborartories, Inc (Andover, MA). Palmitic acid (1-^14^C) and oleic acid (1-^14^C) were purchased from Elmer (Waltham, MA)

### Western blot analysis

LNCaP and LNCaP-AR-V7-pHage cells were lysed by four rounds of freeze/thaw treatment using 1X Reporter Lysis Buffer (Promega) containing 0.4 M NaCl. A 7.5% SDS gel was used to resolve 20 μg of protein. Proteins were then detected using the previously described AR441 antibody [48] at a 1:1000 dilution, β-tubulin antibody (Upstate, Lake Placid, NY) at a 1:10,000 dilution, p-AMPK antibody (Cell Signaling, Danvers, MA) at 1:2000 dilution, AMPK antibody (Cell Signaling, Danvers, MA) at 1:2000 dilution, and ECL reagents (GE Healthcare). ImageJ densitometry software was used to quantify protein expression.

### Quantitative RT-PCR

TRIzol reagent (Invitrogen) was used to prepare RNA from the treated LNCaP and LNCaP-AR-V7-pHage cells. cDNA Synthesis Master Mix (GenDEPOT) was used to reverse transcribe the total RNA (1 μg) to make cDNA to measure target genes. Target gene expression was analyzed using SYBR green PCR Master mix and an ABI 7500 Fast sequence detection system. Previously described PSA, FKBP5, and 18S primer sets were used [47, 49]. The primer set for MDH1 (malate dehydrogenase 1) is sense 5′-TTTCCACCTTGCGGGGTATG-3′ and antisense 5′-TGATTGGTTCAGACTTATCGTCG-3′. The primer set for OGDH (α-ketoglutarate dehydrogenase 1) is sense 5′-­­­CGCTCATCAGGGCATATCAGA-3′ and antisense 5′-CCAGGCCATAGAACCCAAGT-3′. The primer set for GLUD1 (glutamate dehydrogenase 1) is sense 5′-ATCCTGCGGATCATCAAGCC­­­-3′ and antisense 5′-AACGGATACCTCCCTTGCAG-3′. The primer set for ACACA (acetyl-CoA carboxylase alpha) is sense 5′-GTCTCGGCCCTGCTTTACTA­­­-3′ and antisense 5′-CTGATTTGGGGATCTCTAGCC-3′. The primer set for ELOVL7 (fatty acid elongase L7) is sense 5′-­­­TCTATGAATCCTTGAGGGCCTA-3′ and antisense 5′-TGACAACATCCACAGAATGTTCC-3′. The primer set for FASN (fatty acid synthase) is sense 5′-­­­ACGATGACCGTCGCTGGAAG-3′ and antisense 5′-GAATCTGGGTTGATGCCTCCG-3′.

### Growth assay

LNCaP and LNCaP-AR-V7-pHage cells (50,000-70,000 cells/well of a 6-well plate) were changed to RPMI 10% charcoal stripped medium and treated with vehicle (EtOH), 1 nM R1881, or 20 ng/mL of doxycycline (Dox) for six days. Medium and treatments were replenished on day 3. Cells were harvested and counted utilizing a Coulter Counter as previously described [50].

### Cell migration assay

LNCaP and LNCaP-AR-V7-pHage cells were changed to 10% charcoal stripped medium and treated with vehicle (EtOH), 1 nM R1881, or 20 ng/mL of Dox for 24 hours. Then the cells were changed to serum-free RPMI media overnight. Then 150,000 cells were plated in serum-free RPMI media treated with either vehicle (EtOH), 1 nM R1881, or 20 ng/mL Dox into the top chamber of BD Falcon 24-well multi-well plate with cell inserts (BD Bioscience, Franklin Lakes, NJ). The bottom chamber had full medium to serve as a chemo-attractant for the cells to migrate towards. After 48 hours the cells were washed with PBS, fixed with cold 100% methanol, stained with crystal violet, imaged under the microscope, and then counted.

### Seahorse assay

ECAR (extracellular acidification rate), a measure of the rate of glycolysis and oxygen consumption rate (OCR), a measure of the rate of OXPHOS, were determined using a Seahorse Bioscience XF24 analyzer (Seahorse Bioscience, North Billerica, MA, USA). LNCaP and LNCaP-AR-V7-pHage cells were plated at 30,000 cells/well in Seahorse XF24 plates coated with BD Cell-Tak (BD Biosciences, Bedford, MA, USA) and incubated overnight. Cells were then changed to RPMI 10% stripped media and treated with vehicle (EtOH), 10 nM R1881, or 20 ng/mL Dox for 24 hours. Basal measurements and measurements in the presence of several inhibitors: oligomycin (1.25 μM), carbonyl cyanide 4-(trifluoromethoxy)phenylhydrazone (FCCP) (1.25 μM), and rotenone (1.25 μM) from XF Cell Mito Stress Kit (Seahorse Bioscience) were taken and normalized to cell number.

### Metabolomics

LNCaP and LNCaP-AR-V7-pHage cells were plated at 6 million cells per 10 cm plate and then changed to RPMI 10% stripped media and treated with vehicle (EtOH), 10 nM R1881, or 20 ng/mL Dox for 48 hours. All parental LNCaP cells received Dox treatment to control for any non-specific effects due to Dox treatment. After 48 hours, cells were counted and 5 million cells from each plate were centrifuged to control for cell number. Four biological replicates collected over several, independent weeks were prepared for each condition. Mass spectrometry-based metabolomics profiling using Liquid Chromatography/Mass Spectrometry (LC/MS) was done as previously described [30, 51].

### Metabolic flux analysis

LNCaP and LNCaP-AR-V7-pHage were plated at 6 million cells per 10 cm plate and then changed to RPMI 10% stripped medium and treated with vehicle (EtOH), 10 nM R1881, or 20 ng/mL Dox for 24 hours. All parental LNCaP cells also received Dox treatment to control for any non-specific effects of Dox on the metabolites. Then medium was changed to starvation medium (serum-free RPMI lacking either glucose or glutamine) for 6 hours. Cells were then transferred to either RPMI 10% dialyzed FBS with either [U-13C]-glucose (11 mM), [1-13C]-glutamine (2 mM), or [5-13C]-glutamine (2 mM) for 0 and 3 hours. After the incubation period, medium was removed and the cells were gently washed with PBS, flash-frozen with liquid nitrogen, harvested with a mixture of water and methanol (1:4), and stored at −80°C.

For processing, the frozen samples were thawed and then mixed with 600 μl of ice-cold chloroform. The resulting homogenate was then mixed with 260 μl of ice-cold water and vortexed again for 2 min. The homogenate was incubated at −20°C for 20 min and centrifuged at 4°C for 10 min to partition the aqueous and organic layers and remove the protein at the interface. The aqueous and organic layers were combined and dried at 37°C for 45 min in an automatic Environmental Speed Vac® system (Thermo Fisher Scientific, IL). The extract was reconstituted in 500 μl of ice-cold methanol: water (1:1) and filtered through a 3 KDa molecular filter (Amicon Ultracel-3K Membrane, Millipore Corporation) at 4°C for 90 min to remove proteins. The filtrate was dried at 37°C for 45 min in a speed vacuum and stored at −80°C until mass spectrometry analysis. Prior to mass spectrometry analysis, the dried extract was re-suspended in 50 μL of methanol:water (1:1) containing 0.1% formic acid and then analyzed using SRM (selected reaction monitoring). Ten microliters were injected and analyzed using a 6490 QQQ triple quadruple mass spectrometer (Agilent technologies) coupled to a 1290 series HPLC system via SRM. Metabolites were targeted in both positive and negative ion mode, ESI voltage was +4000 V in positive ion mode and −3500 V in negative ion mode. Approximately 9-12 data points were acquired per detected metabolite. Samples were delivered to the MS via normal phase chromatography using a Luna Amino column (4um, 100A 2.1×150mm, Phenominex) at 400 μl/min gradient spanning 80 % B to 2 % B over a 20 minute period followed by 2% B to 80% B for a 5 min period and followed by 80% B for 8 minute time period re-equilibrate the column. Buffer A was comprised of 5 mM ammonium acetate (pH = 9.9) in water and Buffer B was 100% acetonitrile. To assess the validity of our method for calculating isotopomers, we determined the complete isotopomer distributions for each metabolite.

Data analysis was performed in Quantitative Analysis (Agilent MassHunter (Santa Clara, CA)) and estimated the % of isotopomer incorporation using the formula [% of Incorporation = ^13^C/^13^C+^12^C) X100] and was corrected for isotopomeric distribution and natural abundance.

### Oil Red O staining

LNCaP and LNCaP-AR-V7-pHage cells were plated at 50,000 cells per well in a 6-well plate and allowed to adhere overnight in a humidified 5% CO_2_ incubator at 37°C. The next day, the medium was changed to stripped serum and the cells were treated with either EtOH, 10 nM R1881 or 20 ng/mL Dox for 72 hours. After this initial incubation, the media and treatment were changed out and replaced with a fresh set of stripped media and treatment for another 72 hours for a total treatment time of six days. When harvesting the cells, the medium was aspirated, and the cells were gently washed with PBS. Next the cells were stained with Oil Red O for 15 minutes at room temperature. After the stain was removed, the cells were washed with water and then imaged under a Nikon Phase Contrast 2 ELWD 0.3 microscope at 20X magnification.

### Radiolabeled CO_2_ trap assays

LNCaP and LNCaP-AR-V7-pHage cells were plated in 24-well CellBIND (Corning, Manassas, VA, USA) plates at a density of 60,000 cells per well in RPMI 10% FBS medium. The next day the cells were changed to 10% charcoal stripped medium and treated with vehicle (EtOH), 10 nM R1881, or 20 ng/mL of Dox for 24 hours. After 24 hours, radiolabelled palmitate or oleate was added for 3 hours. Then 50 μL perchloric acid was added and allowed to incubate for 1.5 hours. Lastly, ^14^CO_2_ was collected in CO_2_ traps and counted using a scintillation counter [16].

### Statistical analysis

All data for the experiments were presented as mean ± SEM. The mean displayed was the average of at least triplicate biological samples in each experiment. Each experiment was repeated at least three independent times with the exception of the metabolomics and metabolic flux assays. Both student's t-test and one-way ANOVA followed by a Tukey post-hoc statistical analysis were used to analyze the results using GraphPad PRISM. Differences were considered to be statistically significant at p < 0.05.
